# *PAX6* aniridia and interhemispheric brain anomalies

**Published:** 2009-10-17

**Authors:** Hana Abouzeid, Mohamed A. Youssef, Nihal ElShakankiri, Philippe Hauser, Francis L. Munier, Daniel F. Schorderet

**Affiliations:** 1 Jules-Gonin Eye Hospital, University of Lausanne, Switzerland; 2 IRO – Institute for Research in Ophthalmology, Sion, Switzerland; 3 Department of Radiology, CHUV, University of Lausanne, Switzerland; 4 Department of Paediatrics, Genetics Unit, University of Alexandria, Egypt; 5 Department of Ophthalmology, Paediatric Unit, University of Alexandria, Egypt; 6 EPFL - Ecole polytechnique fédérale de Lausanne, Lausanne, Switzerland

## Abstract

**Purpose:**

To report the clinical and genetic study of patients with autosomal dominant aniridia.

**Methods:**

We studied ten patients with aniridia from three families of Egyptian origin. All patients underwent full ophthalmologic, general and neurological examination, and blood drawing. Cerebral magnetic resonance imaging was performed in the index case of each family. Genomic DNA was prepared from venous leukocytes, and direct sequencing of all the exons and intron–exon junctions of the Paired Box gene 6 (*PAX6*) was performed after PCR amplification. Phenotype description, including ophthalmic and cerebral anomalies, mutation detection in *PAX6* and phenotype-genotype correlation was acquired.

**Results:**

Common features observed in the three families included absence of iris tissue, corneal pannus with different degrees of severity, and foveal hypoplasia with severely reduced visual acuity. In Families 2 and 3, additional findings, such as lens dislocation, lens opacities or polar cataract, and glaucoma, were observed. We identified two novel (c.170-174delTGGGC [p.L57fs17] and c.475delC [p.R159fs47]) and one known (c.718C>T [p.R240X]) *PAX6* mutations in the affected members of the three families. Systemic and neurological examination was normal in all ten affected patients. Cerebral magnetic resonance imaging showed absence of the pineal gland in all three index patients. Severe hypoplasia of the brain anterior commissure was associated with the p.L57fs17 mutation, absence of the posterior commissure with p.R159fs47, and optic chiasma atrophy and almost complete agenesis of the corpus callosum with p.R240X.

**Conclusions:**

We identified two novel *PAX6* mutations in families with severe aniridia. In addition to common phenotype of aniridia and despite normal neurological examination, absence of the pineal gland and interhemispheric brain anomalies were observed in all three index patients. The heterogeneity of *PAX6* mutations and brain anomalies are highlighted. This report emphasizes the association between aniridia and brain anomalies with or without functional impact, such as neurodevelopment delay or auditory dysfunction.

## Introduction

In the majority of cases, aniridia is a panophthalmopathy that is characterized by the absence or hypoplasia of the iris and is associated with other ocular anomalies, such as cataract, foveal hypoplasia, corneal opacity (pannus), coloboma, anterior chamber angle (with secondary glaucoma), or optic nerve malformations, mainly hypoplasia (OMIM 106210). Mostly inherited in an autosomal dominant mode, aniridia can have variable expressivity, and about one-third of the patients are sporadic cases. Mutations in the Paired Box gene 6 (*PAX6*) located on chromosome 11p13 [1] have been shown to cause aniridia [2]. *PAX6* encodes a transcription factor essential for the development of the structures and axes of the eye [3]. Knocking out *PAX6* from the mouse genome results in the absence of the eye, the so-called Small eye (Sey) mouse [4,5].

Furthermore, *PAX6* plays a major role in brain development [6,7] where it is expressed in the telencephalon, the diencephalon, the caudal part of the rhombencephalon, the myelencephalon, and the spinal cord [3,8]. The early death of a baby with a compound heterozygous *PAX6* mutation has been reported, and the brain of this child displayed major brain and cytoarchitectonic abnormalities [9]. Brain imaging (magnetic resonance imaging [MRI]) performed in patients heterozygous for *PAX6* mutations often reveals various malformations as well. Absence of the brain anterior or posterior commissure, absence of the pineal gland, and a present but reduced in size corpus callosum have all been reported [10-14]. These anomalies involve the brain interhemispheric fibers and can thus affect the auditory functions that depend on these fibers [10].

Genotype–phenotype correlations have shown that mutations causing premature termination codons are associated with aniridia and missense mutations are related to non-aniridia phenotypes, such as isolated foveal hypoplasia, microphthalmia, and optic nerve defects [15].

The aim of this study was to analyze three families of Egyptian origin, to describe their clinical phenotype, including brain imaging, and to report the results of their molecular screening. We present two novel and one known nonsense mutations associated with brain anomalies.

## Methods

**Figure 1:**
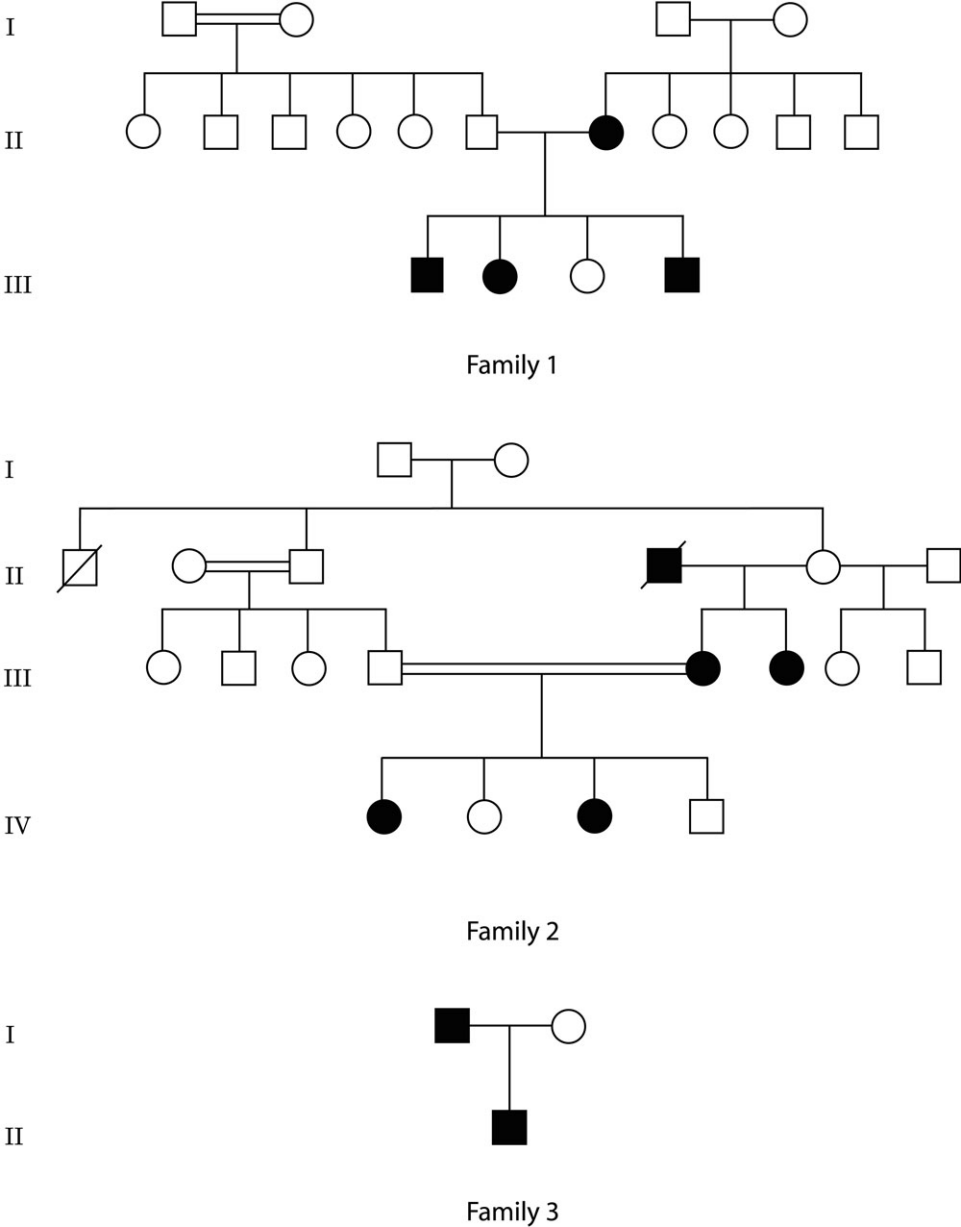
Pedigrees and mutation sequences of the three Egyptian families with autosomal dominant aniridia. Male and female subjects are represented by squares and circles, respectively, and affected family members have darkened symbols.

This study was approved by the Ethics Committee of the Faculty of Medicine of the University of Alexandria, Egypt, and was conducted in accordance to the tenets of the Declaration of Helsinki. Written informed consent was obtained from each participant or parent. Ten patients with aniridia belonging to three families of Egyptian origin were included in this study as well as six first-degree relatives (Figure 1). The three families were from the Governorate of Alexandria, in northwestern Egypt. All subjects underwent full ophthalmic, general, and neurological examination, respectively, at the Departments of Ophthalmology, Pediatrics and Neurology of the University of Alexandria, Egypt. Special attention was paid to assessing the presence of associated anomalies, such as Wilms’ tumor, urogenital anomalies, or mental retardation, in all subjects. We performed a cerebral MRI in each index patient (Patient III-1 of Family 1, IV-3 of Family 2, and I-1 of Family 3; Table 1). MRIs were reviewed by two different radiologists. MRI acquisition techniques included conventional T1- and T2-weighted multisection images (5-mm slice) on a GE Signa HDx 1.5Tesla MRI (General Electric Company, Fairfield, CT).

**Table 1:** Ocular and cerebral assessment of the ten patients with aniridia.

**Patient number**	***PAX6* Mutation**			**Brain MRI findings**	**Age at examination**	**Visual Acuity RE/LE**	**Corneal pannus**	**Glaucoma**	**Lens status**
	**Exon**	**DNA**	**Protein**						
Family 1									
II-7	7	c.682-686delTGGGC*	Q57fx17	NA	46y	0.1/0.15	yes	yes, bilateral trabeculectomy	bilateral aphakia
III-1	7	c.682-686delTGGGC*	Q57fx17	absent pineal gland, anterior commissure severe hypoplasia	19y	0.05/0.05	no	yes, bilateral trabeculectomy	bilateral anterior polar cataract with peripheral lens opacities, superior dislocation
III-2	7	c.682-686delTGGGC*	Q57fx17	NA	17y	0.05/0.05	yes	no	pseudophakic
III-4	7	c.682-686delTGGGC*	Q57fx17	NA	11y	0.05/0.05	mild	no	faint cortical opacities, superior dislocation
Family 2									
III-5	8	g.30586227delC*	R159fx47	NA	36y	0.1/0.2	yes	no	bilateral anterior polar cataract, faint cortical opacities
III-6	8	g.30586227delC*	R159fx47	NA	44y	HM/LP	severe	yes, uncontrolled	superior dislocation
IV-1	8	g.30586227delC*	R159fx47	NA	10y	0.1/0.1	no	no	bilateral aphakia
IV-3	8	g.30586227delC*	R159fx47	absent pineal gland, absent posterior commissure	17y	0.1/0.1	yes	no	bilateral posterior polar cataract, superior dislocation
Family 3									
I-1	9	g.30579567C>T	R240X	absent pineal gland, optic chiasma atrophy, almost complete agenesis of the corpus callosum	35y	0.1/0.1	mild	no	normal
II-1	9	g.30579567C>T	R240X	NA	3m	NA	no	no	normal

The asterisk indicated a novel mutation, RE: right eye, LE: left eye, NA: not available.

### Molecular analyses

DNA from patients was extracted from peripheral leucocytes, as previously described [16]. Direct bidirectional resequencing of all PCR-amplified coding exons and adjacent junctions was performed with the ABI Dye Terminator, version 1 (Applied Biosystems, Foster City, CA), in a final reaction volume of 10 μl, and electrophoresed on a 3130XL ABI genetic analyzer (Applied Biosystems). Sequences were compared to the reference sequence NM_000280.3, using Chromas version 2.23 (Technelysium, Tewantin, Australia). The adenine of the ATG translation start site was set to 1. Primers and annealing temperature for *PAX6* exons are listed in Table 2. In short, amplification was performed in a thermal cycler (GeneAmp 9700; Applied Biosystems), in a total volume of 30 μl. Each PCR contained 100 ng genomic DNA, 0.9 nl of each primer, and 15 μl master mix 2× (Qiagen, Hombrechtikon, Switzerland), with or without betaine. PCR reactions were performed as follows: an initial denaturation step was carried out for 10 min followed by 35 cycles of 1 min at 92 °C, 1 min at the specific annealing temperature (Table 2), and 1 min at 72 °C. A final extension cycle at 72 °C for 10 min was performed. Identified mutations were evaluated in 96 ethnic-matched controls by denaturing high-performance liquid chromatography (DHPLC) on a WAVE system (Transgenomics, Crewe, UK). Buffer A contained 0.1 M triethylammonium acetate (TEAA; Transgenomics). Buffer B contained 0.1 M TEAA and 25% acetonitrile HPLC grade (Sigma-Aldrich Co., St. Louis, MO). The flow rate was set at 1.5 ml/min, and the Buffer B gradient was increased by 5% per min for 2 min. The optimum temperature was determined by the Wavemaker software (Transgenomic) for each DNA fragment, and a time shift was applied as needed (Table 2). Initial Buffer B concentrations and temperatures for each fragment are listed in Table 2.

**Table 2:** List of initial buffer B concentrations and temperatures for each DNA fragment.

**PCR**				**WAVE**	
**Exon**	**Sense primer (5’-3’)**	**Antisense primer (5’-3’)**	**Annealing temperature °C**	**Temperature °C**	**% start B**
1	TGTTGCGGAGTGATTAGTGG	TCCTGGGAAGGAGACAGAGA	60 +betain	60.8	57.9
2	ACACACTTGAGCCATCACCA	CTCCTGCGTGGAAACTTCT	60 +betain	59.3	60.6
3	GTGGGTGTAATGCTGGGACT	CCCAATCTGTTTCCCCTACA	60 +betain	56	59.7
4	CCCCAAGAGGTTGAGTGGAT	GTCGCGAGTCCCTGTGTC	60 +betain	61.4	57.1
5	TGAGGATGCATTGTGGTTGT	GTGGAAGGAGAGGGGAAAGT	60 +betain	59.5	60.8
6	TTCAGGCAGTGTTTAAGAAAAGTT	ACTCACACATCCGTTGGACA	55	54.3	58.7
7	TGCAGATGCAAAAGTCCAAG	CTCTGTTCCCCCAGGTACAA	60 +betain	57.4	61
8	TTTCCACGGTGTATCTGCAA	AAGCCCTGAGAGGAAATGGT	60 +betain	59.6	59.9
9	ACCTTGGGAATGTTTTGGTG	CACTGAAAAGATGCCCAGAGA	60 +betain	56.7	60.3
10	AGGTGGGAACCAGTTTGATG	CATGGCAGCAGAGCATTTAG	60 +betain	57.5	58
11	TTCAGTCTGCTAAATGCTCTGC	TGTGAGGGCTGTGTCTGTTC	60 +betain	59	60
12	ACCACACCGGGTAATTTGAA	CTCTCAAGGGTGCAGACACA	60 +betain	58.7	58.4
13	TAGCTCGAGGCCCAATCTTA	TAAACACGCCCTCCCATAAG	60 +betain	58.3	60
14	TTTCTGAAGGTGCTACTTTTATTTG	AAGTCCATTCCTTCCCCAGT	55	53.5	60.2
15	AAACTTAAGTGTTTTGAAGTTGTTCAC	CCCAGATTGAAAATGCCAGT	60 +betain	54.3	60.5

## Results

### Clinical findings

**Figure 2:**
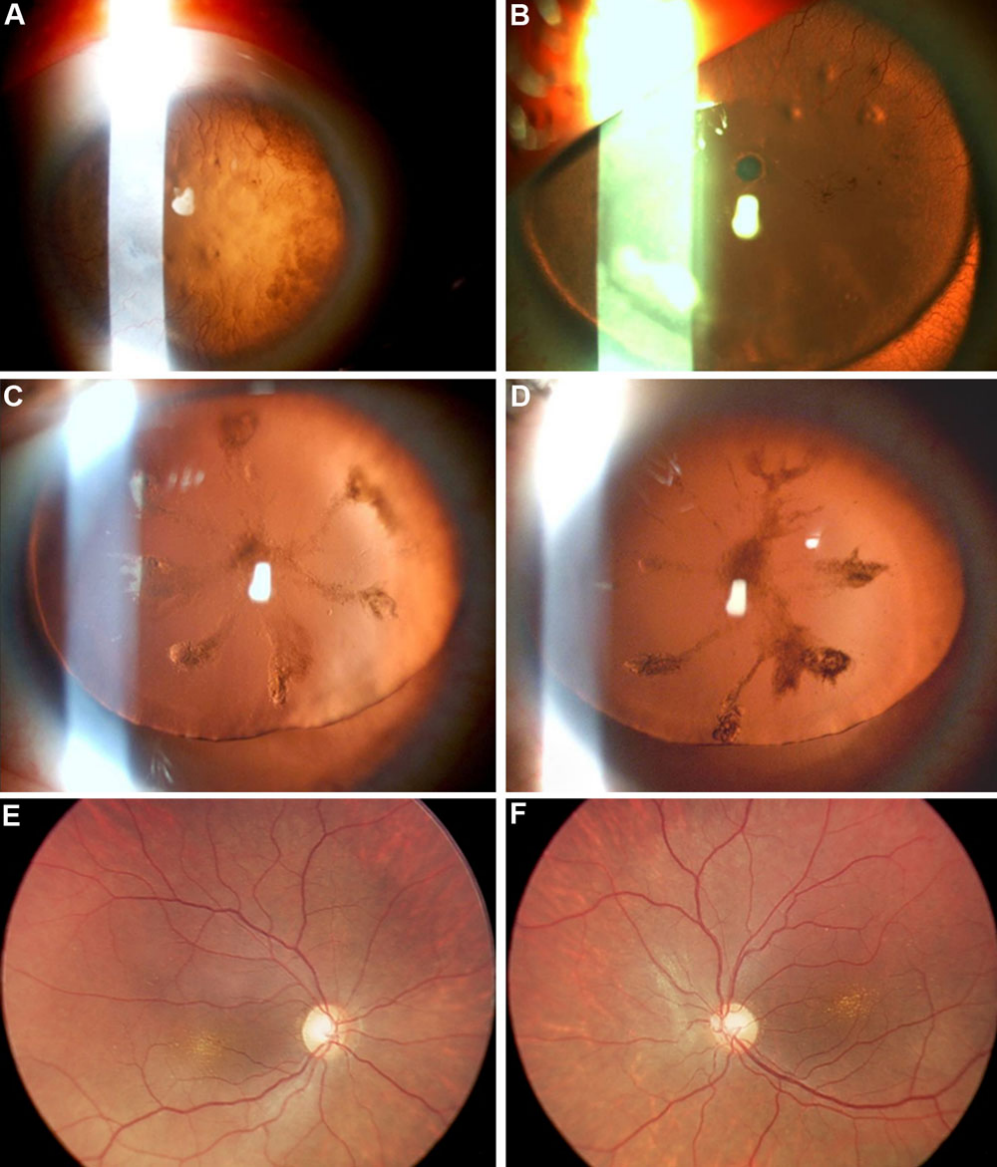
Slit-lamp photographs. **A**: Right eye of patient II-7 from Family 1. Note the significant, heavy, corneal vascularization sparing the nasal area. The iris base is very thin, almost invisible, a typical feature of aniridia. The patient was aphakic since cataract surgery performed in childhood. Best-corrected visual acuity was 0.1. **B**: Right eye patient III-4 from Family 1 showing heavy corneal vascularization (pannus) and superior dislocation of the lens. Almost no iris residual tissue is visible, as typically seen in aniridia. Note as well the small anterior polar cataract and the associated faint peripheral cortical opacities. **C** and **D**: Right and left eye of patient IV-3 from Family 2. Note the bilateral posterior polar cataract shaped like the petals of a flower and the superior lens dislocation. **E** and **F**: Fundus photographs. Right and left eye of patient IV-1 from Family 2. Foveal hypoplasia is observed with macular pigment epithelium alterations.

Patients clinical features and mutations description are detailed in Table 1. All ten affected patients from the three families had bilateral aniridia, with almost complete absence of iris tissue at its base, and bilateral foveal hypoplasia. Pendular nystagmus consecutive to early severely reduced visual acuity was observed in all affected subjects, except patient III-6 of Family 2 (Figure 1) who presented a left exotropia. Corneal vascularization of different degrees was observed in the three families (Figure 2). Lens abnormalities and glaucoma were present in Families 1 and 2 only. In Family 1, the two affected subjects with glaucoma had controlled pressure after bilateral trabeculectomy, whereas patient III-6 of Family 2 had uncontrolled high pressure. Systemic and neurological examinations were normal in all ten affected subjects. In particular, no kidney or urogenital anomalies on ultrasonography, no mental retardation, no cerebellar abnormal signs, and no olfactory or hearing difficulties were observed or reported.

**Figure 3:**
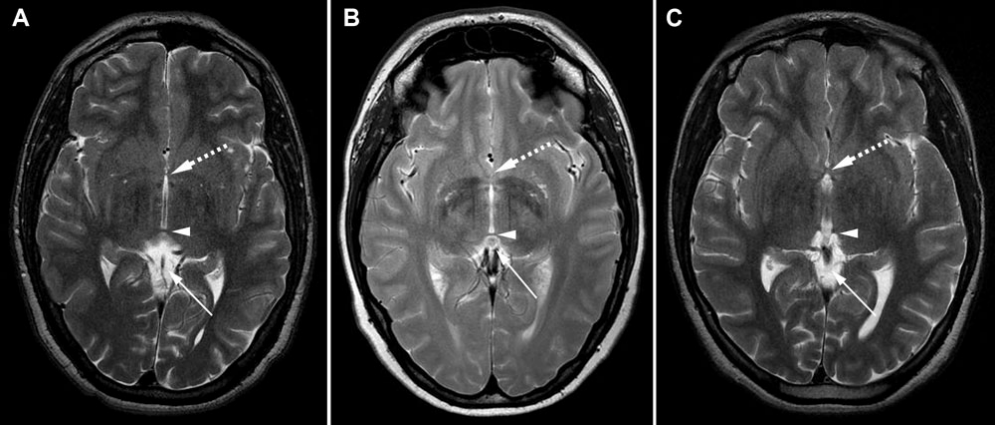
Axial cerebral T2-weighted magnetic resonance images. **A**: Patient III-1 from Family 1. Dashed arrow: severe hypoplasia of the anterior commissure. Arrow head: normal posterior commissure. Lower arrow: absence of the pineal gland. **B**: Normal magnetic resonance imaging (MRI) images. Dashed arrow: normal anterior commissure. Arrow head: normal posterior commissure. Lower arrow: normal pineal gland. **C**:  Patient IV-3 from Family 2. Dashed arrow: normal anterior commissure. Arrow head: absent posterior commissure. Lower arrow: absent pineal gland.

**Figure 4:**
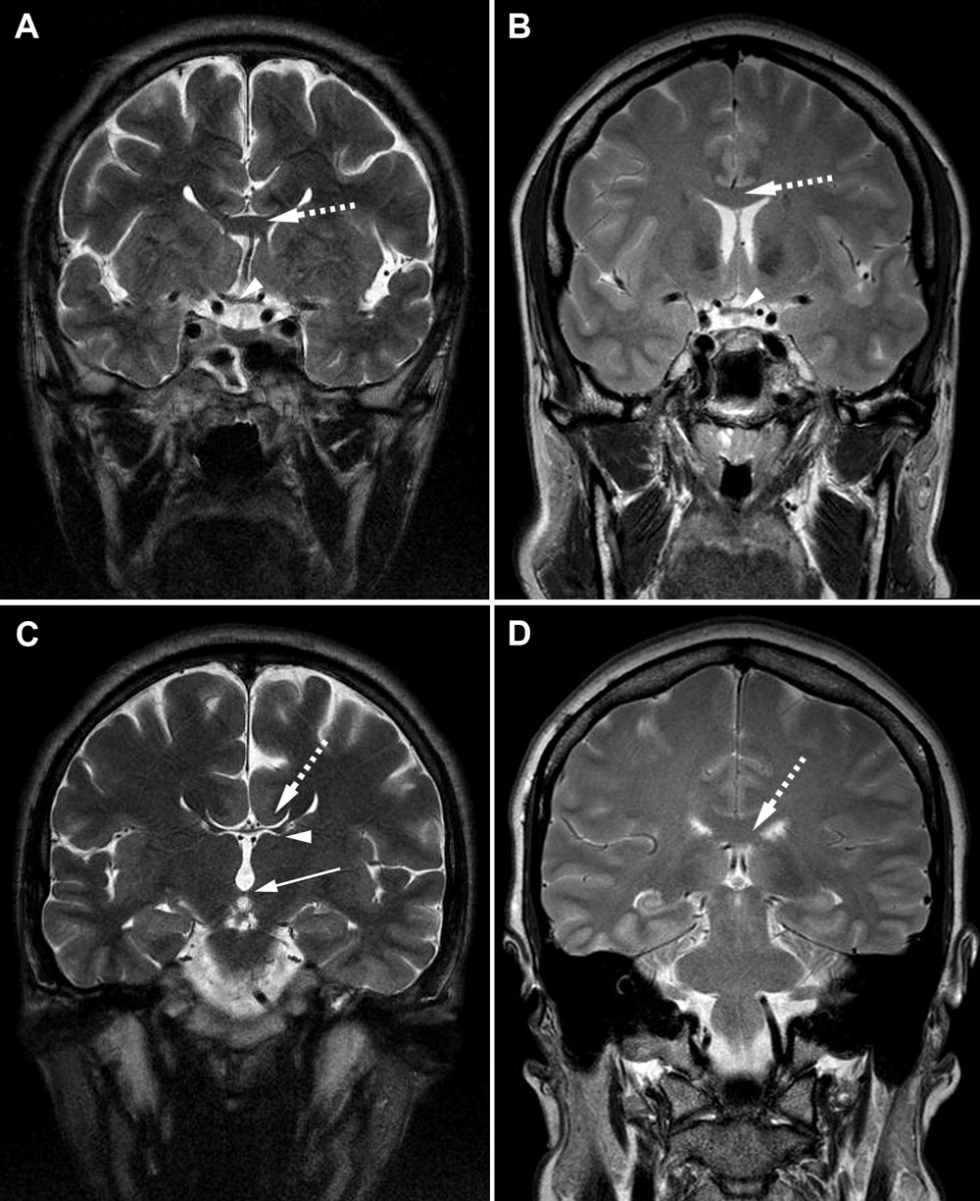
Coronal cerebral T2-weighted magnetic resonance images. **A**: Patient I-1 from Family 3. Dashed arrow shows severe hypogenesis of the corpus callosum with small amount of remnant tissue localized at the virtual connection between the genu and the body of the corpus callosum; arrow head shows the atrophic optic chiasm. **B**: Normal MRI images. Dashed arrow shows normal corpus callosum and arrow head shows normal optic chiasm. **C**: Patient I-1 from Family 3. Dashed arrow shows lateral callosal bundles of Probst, which are hemispheric connection fibers that did not cross the midline and that are seen in callosal dysgenesis. Superomedial margins of the lateral ventricles are indented by the Probst bundles. Arrow head shows remnants of the corpus callosum. Lower arrow shows normal posterior commissure. **D**: Normal MRI image with dashed arrow pointing normal corpus callosum.

Cerebral MRI of Patients III-1 of Family 1 showed a severe hypoplasia of the anterior commissure of the brain and an absent pineal gland; the posterior commissure of the brain was normal (Figure 3A). In Patient IV-3 of Family 2, a total absence of both the pineal gland and the posterior commissure of the brain was observed, but the anterior commissure was normal (Figure 3C). Patient I-1 of Family 3 harbored an almost complete agenesis of the corpus callosum with a small amount of remnant tissue localized at the virtual connection between the genu and the body of the corpus callosum (Figure 4A). In the same patient, Probst bundles were identified (Figure 4C). Concomitant ectasia of the ventricles and atrium, hypoplasia of the optic chiasm (Figure 4C), and total absence of the pineal gland were observed as well (Figure 4C). Both anterior and posterior commissures of the brain were normal.

### Molecular findings

In Family 1, a c.170-174delTGGGC mutation was identified in exon 7, generating a frameshift and an early termination 17 codons downstream (p.L57fs17). The mutation was present at the heterozygous state in the four affected members of Family 1 and absent in the two unaffected subjects (Figure 1). Family 2 also exhibited an unreported deletion. The deletion of c.475delC in exon 8 generates a frameshift and a new stop codon 47 amino acids downstream (p.R159fs47). This mutation was present in the three affected subjects and absent in the two unaffected individuals (Figure 1). The two mutations were not detected in 96 ethnically matched healthy individuals and have not been previously reported according to the Human PAX6 Mutation Database [17]. In Family 3, the previously described c.718C>T (p.R240X) nonsense mutation in exon 9 [2,18,19] was detected in the two affected members and not in the unaffected mother (Figure 1).

## Discussion

We report three *PAX6* mutations segregating in three families with aniridia originating from the northwestern part of Egypt. Two of the three mutations are novel frameshifting deletions, and one is a previously described nonsense mutation. To our knowledge, this is the first report of aniridia mutations from this part of the world. It is interesting to note that the pathology of the *PAX6-*related genome is far from being fully determined when one considers that only one of the identified mutants was already known. From a clinical point of view, the ten patients of this series harbored common but extensive features of ocular-isolated aniridia associated with the wide spectrum of already described MRI brain malformations. These include absence of the pineal gland and hypogenesis of the corpus callosum and of the anterior or posterior commissure. The interpretation of the observed MRI malformations is limited by the lack of a full assessment of the neurological development; refined examinations, such as electroencephalogram, neurocognitive tests, or sleep study, were not available. Nevertheless, the patients did not harbor major neurodevelopmental delay that could be detected clinically by a senior geneticist and pediatrician.

In a large genotype–phenotype correlation of the *PAX6* Mutation Database, Tzoulaki et al. [15] established that mutations introducing a premature termination codon (PTC) were predominantly associated with aniridia, whereas non-aniridia phenotypes, such as isolated foveal hypoplasia, microphthalmia, and optic nerve defects, were predominantly caused by missense mutations. These authors showed that the second most frequent type of *PAX6* mutations was frameshifting insertions or deletions and missense mutations were the third. In the present series of patients with typical aniridia, the three identified mutations caused a PTC, and two of them were frameshifting deletions, thus confirming the frequent association of PTC and of frameshifting mutations with aniridia [15].

The p.R240X mutation segregating in Family 3 has previously been described [2,18,19] with more than 20 independent records in the *PAX6* Mutation Database [17]. This mutation is a C>T transition that occurs on a CpG dinucleotide, a structure known for its high mutability [20]. This CpG in exon 9, located in the homeobox coding region, is a mutation hotspot since CpG dinucleotides of the last third of *PAX6* tend to be methylated and thus more inclined to undergo spontaneous deamination of cytosine, resulting in C>T transition [21]. A sense-strand deamination of CpG in a CGA codon creates a termination codon CTA. It has been proposed that nonsense-mediated decay (NMD), the mechanism responsible for the elimination of mRNAs that contain premature termination codons before the last exon [22], is highly involved in aniridia since the majority of aniridia *PAX6* mutations introduce premature termination codons [15]. Thus, NMD impedes truncated protein formation, and loss-of-function of one allele is responsible for the development of aniridia through haploinsufficiency. This mechanism may explain how different mutations and different putative truncated proteins can induce similar phenotypes. The Family 1 and 2 unreported mutations, p.L57fs17 and p.R159fs47, are both likely to result in haploinsufficiency through NMD.

Hypoplasia of the anterior commissure [10,12,14], of the posterior commissure [10], and of the corpus callosum [10-12,14] have been reported in heterozygous carriers of *PAX6* mutations. Absence or hypoplasia of the anterior commissure is present in up to one-third of *PAX6* mutation carriers [10,13]. Abnormal anterior MRI anomalies can be associated with subtle neurological deficits, such as olfactory difficulties [14], hearing difficulties [10], and deficits in executive and social cognition [11], or with aniridia only [12]. We did observe anomalies of these three brain structures in our three imaged patients (Table 1). Although no brain anomalies can yet be directly related to any specific *PAX6* mutation, we report the third observation of MRI anomalies associated with the p.R240X mutation [10,14]. In contrast with both Sisodiya et al. [14] and Bamiou et al. [10] who reported a hypoplastic anterior commissure but a normal corpus callosum, we observed in Patient I-1 of Family 3 an almost complete agenesis of the corpus callosum with a normal anterior commissure (Table 1, Figure 4C). Interestingly, we observed Probst bundles in this patient (Figure 4C), which highlights the severity of the corpus callosum hypogenesis in this case. Indeed, Probst bundles represent fiber tracts that grow caudally along the medial surface of the ipsilateral cerebral hemisphere that would have crossed the midline in the case of normal corpus callosum development [23]; their presence is common in patients with corpus callosum hypogenesis and twice as frequent in patients with corpus callosum agenesis [24].

We hypothesize that a specific mutation can cause brain anomalies but that the brain anomaly can be expressed differently. Being a transcription factor, *PAX6* interacts with several brain developmental genes and transcription factors, such as the Homeobox gene expressed in ES cells, the *Hesx1* gene [3], whose interaction with *PAX6* could be altered by the presence of a mutant protein causing corpus callosum and brain commissure hypogenesis. It has recently been demonstrated in mouse that the transcription factors Empty spiracles homeobox 2, *Emx2* and *Pax6* are essential for cortical regionalization at the beginning of neuronogenesis [25]. From this perspective, one can hypothesize that abnormal interaction between mutated PAX6 protein and a normal EMX2 protein could be responsible for the presence of the interhemispheric brain anomalies. Moreover, the possibility of digenism is not excluded with the presence of an undetected *EMX2* mutation added to the *PAX6* one to result in corpus callosum and commissure dysgenesis. Last, the existence of at least two promoters is described in the literature to mediate *Pax6* expression in different tissues [26,27]. Thus, one promoter mediates expression in the brain, and differential *PAX6* transcription through alternate promoter usage could be involved in neural development [28].

The auditory fibers travel through the interhemispheric pathways, and it has been demonstrated that children with *PAX6* mutations and abnormalities of the interhemispheric pathways on MRI harbor reduced auditory capacities even in the presence of normal audiograms [10]. We did not observe any major clinical auditory deficits in any of the ten studied patients, although the three patients with available MRIs showed abnormal interhemispheric pathways. However we cannot conclude on the auditory status since we did not perform any specific tests, including the study of auditory-evoked potentials.

Mitchell et al. [13] published an MRI study of 24 aniridia patients with *PAX6* mutations and found absence of the pineal gland in 13/24 patients (54%). These authors concluded that this observation may be common in aniridia patients. Indeed, we have previously reported absence of the pineal gland in aniridia patients [29] as we do in the three patients of the present series in whom we performed MRI. As previously mentioned, sleep study was not performed and thus we could not assess the functional consequences of the absence of pineal glands. Mice homozygous for mutations in the *Pax6* gene harbor a wide variety of neurodevelopmental abnormalities, including absence of the corpus callosum and pineal gland [30,31].

*PAX6* affects the development and function of the central nervous system and the eye as well as the pancreas and the hypothalamopituitary axis through the hypothalamus [3], which shares with the retina a common embryologic origin—the neural plate. Unfortunately, we were not able to perform electroretinography or endocrine testing to study the effect of the *PAX6* mutations on the retina and hypothalamopituitary axis.

Finally, WAGR syndrome (OMIM 194072), which includes Wilms’ tumor, aniridia, genitourinary anomalies, and mental retardation, is caused by either microscopic or submicroscopic deletion of chromosome 11p13-p12 in a region containing both the Wilms tumor 1, *WT1 *gene and the *PAX6* genes. A contiguous syndrome, the WAGRO syndrome (OMIM 612469), includes the features of WAGR with obesity and is caused by a similar deletion that includes the Brain-derived neurotrophic factor, *BDNF* gene as well. Both syndromes were clinically excluded by normal kidney and urogenital ultrasonography performed on our patients.

In summary, we describe three mutations found in three families from northwestern Egypt, adding two novel mutations to the existing spectrum of *PAX6* mutations. Two of the three mutations are frameshifting small deletions and one is a previously described nonsense mutation. While the ten familial aniridia patients of this series harbored typical features of ocular-isolated aniridia, we observed a wide spectrum of MRI brain malformations, including absence of the pineal gland, hypogenesis of the corpus callosum with Probst bundles, and hypoplasia of the anterior or posterior commissure in patients not harboring major neurological development anomalies. Correlation between phenotype and genotype of *PAX6* mutations is still in its infancy in regard to brain malformations. The wide spectrum of *PAX6*-related anomalies and the fact that aniridia is frequently caused by *PAX6* mutations should prompt physicians facing aniridia to perform an examination of the central nervous system (at least with an MRI), an ultrasonography of the kidney and of the urinary pathways, a study of the kidney functions, and most importantly a complete assessment of the pituitary hormones and hypothalamic-releasing hormones.

## References

[1] Mannens M Bleeker-Wagemakers EM Bliek J Hoovers J Mandjes I van Tol S Frants RR Heyting C Westerveld A Slater RM Autosomal dominant aniridia linked to the chromosome 11p13 markers catalase and D11S151 in a large Dutch family. Cytogenet Cell Genet 1989 52 32 6 2575483 10.1159/000132834

[2] Glaser T Walton DS Maas RL Genomic structure, evolutionary conservation and aniridia mutations in the human PAX6 gene. Nat Genet 1992 2 232 9 1345175 10.1038/ng1192-232

[3] Haubst N, Favor J, Götz M. The Role of Pax6 in the Nervous System during Development and in Adulthood: Master Control Regulator or Modular Function? In: Gerald Thiel. Editor. Transcription Factors in the Nervous System. Weinheim: Wiley-VCH Verlag GmbH & Co; 2006. p. 23-51

[4] Hill RE Favor J Hogan BL Ton CC Saunders GF Hanson IM Prosser J Jordan T Hastie ND van Heyningen V Mouse small eye results from mutations in a paired-like homeobox-containing gene. Nature 1991 354 522 5 1684639 10.1038/354522a0

[5] Quiring R Walldorf U Kloter U Gehring WJ Homology of the eyeless gene of Drosophila to the Small eye gene in mice and Aniridia in humans. Science 1994 265 785 9 7914031 10.1126/science.7914031

[6] Stoykova A Gruss P Roles of Pax-genes in developing and adult brain as suggested by expression patterns. J Neurosci 1994 14 1395 412 8126546 10.1523/JNEUROSCI.14-03-01395.1994 PMC6577564

[7] Jones L López-Bendito G Gruss P Stoykova A Molnár Z Pax6 is required for the normal development of the forebrain axonal connections. Development 2002 129 5041 52 12397112 10.1242/dev.129.21.5041

[8] Norman MG, McGillivray BC, Kalousek DK, Hill A, Poskitt KJ. Congenital Malformations of the Brain. New York: Oxford University Press; 1995

[9] Glaser T Jepeal L Edwards JG Young SR Favor J Maas RL PAX6 gene dosage effect in a family with congenital cataracts, aniridia, anophthalmia and central nervous system defects. Nat Genet 1994 7 463 71 7951315 10.1038/ng0894-463

[10] Bamiou DE Free SL Sisodiya SM Chong WK Musiek F Williamson KA van Heyningen V Moore AT Gadian D Luxon LM Auditory interhemispheric transfer deficits, hearing difficulties, and brain magnetic resonance imaging abnormalities in children with congenital aniridia due to PAX6 mutations. Arch Pediatr Adolesc Med 2007 161 463 9 17485622 10.1001/archpedi.161.5.463

[11] Ellison-Wright Z Heyman I Frampton I Rubia K Chitnis X Ellison-Wright I Williams SC Suckling J Simmons A Bullmore E Heterozygous PAX6 mutation, adult brain structure and fronto-striato-thalamic function in a human family. Eur J Neurosci 2004 19 1505 12 15066147 10.1111/j.1460-9568.2004.03236.x

[12] Free SL Mitchell TN Williamson KA Churchill AJ Shorvon SD Moore AT van Heyningen V Sisodiya SM Quantitative MR image analysis in subjects with defects in the PAX6 gene. Neuroimage 2003 20 2281 90 14683729 10.1016/j.neuroimage.2003.07.001

[13] Mitchell TN Free SL Williamson KA Stevens JM Churchill AJ Hanson IM Shorvon SD Moore AT van Heyningen V Sisodiya SM Polymicrogyria and absence of pineal gland due to PAX6 mutation. Ann Neurol 2003 53 658 63 12731001 10.1002/ana.10576

[14] Sisodiya SM Free SL Williamson KA Mitchell TN Willis C Stevens JM Kendall BE Shorvon SD Hanson IM Moore AT van Heyningen V PAX6 haploinsufficiency causes cerebral malformation and olfactory dysfunction in humans. Nat Genet 2001 28 214 6 11431688 10.1038/90042

[15] Tzoulaki I White IM Hanson IM PAX6 mutations: genotype-phenotype correlations. BMC Genet 2005 6 27 15918896 10.1186/1471-2156-6-27 PMC1156885

[16] Abouzeid H Munier FL Thonney F Schorderet DF Ten novel RB1 gene mutations in patients with retinoblastoma. Mol Vis 2007 13 1740 5 17960112

[17] Human PAX. 6 Mutation Database [Internet]. Edinburgh: MRC Human Genetics Unit. 2007-[cited 2009 Feb 10]. Available from: http://lsdb.hgu.mrc.ac.uk/home.php?select_db=PAX6/

[18] Redeker EJ de Visser AS Bergen AA Mannens MM Multiplex ligation-dependent probe amplification (MLPA) enhances the molecular diagnosis of aniridia and related disorders. Mol Vis 2008 14 836 40 18483559 PMC2375324

[19] Robinson DO Howarth RJ Williamson KA van Heyningen V Beal SJ Crolla JA Genetic analysis of chromosome 11p13 and the PAX6 gene in a series of 125 cases referred with aniridia. Am J Med Genet A 2008 146A 558 69 18241071 10.1002/ajmg.a.32209

[20] Cooper DN Krawczak M Cytosine methylation and the fate of CpG dinucleotides in vertebrate genomes. Hum Genet 1989 83 181 8 2777259 10.1007/BF00286715

[21] Nachman MW Crowell SL Estimate of the mutation rate per nucleotide in humans. Genetics 2000 156 297 304 10978293 10.1093/genetics/156.1.297 PMC1461236

[22] Byers PH Killing the messenger: new insights into nonsense-mediated mRNA decay. J Clin Invest 2002 109 3 6 11781342 10.1172/JCI14841 PMC150830

[23] Probst FP Congenital defects of the corpus callosum. Morphology and encephalographic appearances. Acta Radiol Suppl 1973 331 1 152 4202700

[24] Hetts SW Sherr EH Chao S Gobuty S Barkovich AJ Anomalies of the corpus callosum: an MR analysis of the phenotypic spectrum of associated malformations. AJR Am J Roentgenol 2006 187 1343 8 17056927 10.2214/AJR.05.0146

[25] Muzio L DiBenedetto B Stoykova A Boncinelli E Gruss P Mallamaci A Emx2 and Pax6 control regionalization of the pre-neuronogenic cortical primordium. Cereb Cortex 2002 12 129 39 11739261 10.1093/cercor/12.2.129

[26] Plaza S Saule S Dozier C High conservation of cis-regulatory elements between quail and human for the Pax-6 gene. Dev Genes Evol 1999 209 165 73 10079359 10.1007/s004270050240

[27] Kammandel B Chowdhury K Stoykova A Aparicio S Brenner S Gruss P Distinct cis-essential modules direct the time-space pattern of the Pax6 gene activity. Dev Biol 1999 205 79 97 9882499 10.1006/dbio.1998.9128

[28] Okladnova O Syagailo YV Mössner R Riederer P Lesch KP Regulation of PAX-6 gene transcription: alternate promoter usage in human brain. Brain Res Mol Brain Res 1998 60 177 92 9757029 10.1016/s0169-328x(98)00167-3

[29] Dansault A David G Schwartz C Jaliffa C Vieira V de la Houssaye G Bigot K Catin F Tattu L Chopin C Halimi P Roche O Van Regemorter N Munier F Schorderet D Dufier JL Marsac C Ricquier D Menasche M Penfornis A Abitbol M Three new PAX6 mutations including one causing an unusual ophthalmic phenotype associated with neurodevelopmental abnormalities. Mol Vis 2007 13 511 23 17417613 PMC2649307

[30] Schmahl W Knoedlseder M Favor J Davidson D Defects of neuronal migration and the pathogenesis of cortical malformations are associated with Small eye (Sey) in the mouse, a point mutation at the Pax-6-locus. Acta Neuropathol 1993 86 126 35 8213068 10.1007/BF00334879

[31] Estivill-Torrus G Vitalis T Fernandez-Llebrez P Price DJ The transcription factor Pax6 is required for development of the diencephalic dorsal midline secretory radial glia that form the subcommissural organ. Mech Dev 2001 109 215 24 11731235 10.1016/s0925-4773(01)00527-5

